# Chronic Sleep Deprivation Blocks Voluntary Morphine Consumption but Not Conditioned Place Preference in Mice

**DOI:** 10.3389/fnins.2022.836693

**Published:** 2022-02-17

**Authors:** Darrell Eacret, Crystal Lemchi, Jasmine I. Caulfield, Sonia A. Cavigelli, Sigrid C. Veasey, Julie A. Blendy

**Affiliations:** ^1^Department of Systems Pharmacology and Translational Therapeutics, Perelman School of Medicine, University of Pennsylvania, Philadelphia, PA, United States; ^2^Huck Institute for Life Sciences, Pennsylvania State University, University Park, PA, United States; ^3^Department of Medicine, Center for Sleep and Circadian Neurobiology, Perelman School of Medicine, University of Pennsylvania, Philadelphia, PA, United States

**Keywords:** sleep, reward, morphine, hypothalamus, preference

## Abstract

The opioid epidemic remains a significant healthcare problem and is attributable to over 100,000 deaths per year. Poor sleep increases sensitivity to pain, impulsivity, inattention, and negative affect, all of which might perpetuate drug use. Opioid users have disrupted sleep during drug use and withdrawal and report poor sleep as a reason for relapse. However, preclinical studies investigating the relationship between sleep loss and substance use and the associated underlying neurobiological mechanisms of potential interactions are lacking. One of the most common forms of sleep loss in modern society is chronic short sleep (CSS) (<7 h/nightly for adults). Here, we used an established model of CSS to investigate the influence of disrupted sleep on opioid reward in male mice. The CSS paradigm did not increase corticosterone levels or depressive-like behavior after a single sleep deprivation session but did increase expression of Iba1, which typically reflects microglial activation, in the hypothalamus after 4 weeks of CSS. Rested control mice developed a morphine preference in a 2-bottle choice test, while mice exposed to CSS did not develop a morphine preference. Both groups demonstrated morphine conditioned place preference (mCPP), but there were no differences in conditioned preference between rested and CSS mice. Taken together, our results show that recovery sleep after chronic sleep disruption lessens voluntary opioid intake, without impacting conditioned reward associated with morphine.

## Introduction

Sleep is an essential biological function that has many roles in maintaining the health of an organism, and better sleep quality is beginning to be emphasized in education and medicine (Eban-Rothschild et al., 2017; Ramar et al., 2021). The amount of time adults sleep has decreased since the 1940s, leading the CDC to classify insufficient sleep as a public health epidemic (CDC, 2015). A significant fraction (∼35%) of the US population does not get enough sleep (Liu et al., 2016). Millions of hours and over $400 billion dollars of productivity are lost each year due to the consequences of poor sleep in the United States (Hafner et al., 2017). Beyond economic consequences, the epidemic of poor sleep is concerning as there is an association between sleep duration and risk of death, with those that do not get the recommended 7–9 h of sleep per night at higher mortality risk (Vgontzas et al., 2010; Watson et al., 2015). In addition to risk of death, poor sleep has a plethora of adverse effects on social and emotional behavior (Walker and van der Helm, 2009; Van Der Helm et al., 2010; Gujar et al., 2011; Ben Simon and Walker, 2018), cognitive performance (Dinges, 2005; Walker, 2008), cardiovascular function (Tall and Jelic, 2019; Ahmad and Didia, 2020), as well as analgesic response (Roehrs et al., 2006; Krause et al., 2019) among others.

One adverse consequence of poor sleep is its association with substance use disorders. Specifically, sleep deprivation can increase the vulnerability for drug use (Brower and Perron, 2010). Adolescents are particularly vulnerable to short sleep influencing later drug use (Roane and Taylor, 2008; Sivertsen et al., 2015; Logan et al., 2018). Sleep problems are a predictor of relapse to opioid use in opioid dependent patients in the month after detoxification (Dijkstra et al., 2008). Poor sleep quality is associated with opioid craving in patients going through treatment for opioid dependence in a residential setting (Lydon-Staley et al., 2017). Subjects from inpatient and outpatient settings report poor sleep as a reason for relapse to opioid use (Maulik et al., 2002). These clinical studies highlight the importance of sleep quality as it relates to future opioid use. However, to our knowledge, the impact of chronic sleep disruption on opioid use and reward has not been tested in a controlled preclinical experiment.

Opioid signaling in the brain is influenced by sleep deprivation. Early studies on 72 h REM sleep deprivation generated a short wakefulness period when the rat is placed back in its home cage. The opioid receptor antagonist naloxone decreased the latency to sleep during this period while morphine extended the wake period (Fratta et al., 1987). Sleep deprived rats show decreased mu opioid receptor densities in the limbic system compared to controls (Fadda et al., 1991). Ten days of sleep deprivation in rats increases gene expression of the endogenous opioids proenkephalin and prodynorphin in the striatum (Baldo et al., 2013). Acute sleep deprivation in mice decreased the analgesic properties of morphine in the hotplate test (Alexandre et al., 2017). In humans, worse quality sleep and decreased time sleeping are associated with increased binding potential (BPnd) from [^11^C] Carfentanil at the mu opioid receptor (Campbell et al., 2013). Two nights of sleep disruption (20 min awakening per hour and one random 60 min awakening) decreased the analgesic effect of morphine in healthy adult subjects (Smith et al., 2020). Taken together, these studies show a central interaction between sleep deprivation and opioid signaling.

Consequences of sleep deprivation also include neuroinflammation. Sleep deprivation for both 1 and 7 days increased the expression of Iba-1, a microglial marker, in the mouse brain (Zhu et al., 2012). Chronic short sleep (CSS), 8 h/day, 3x/week for 4 weeks increased Iba-1, GFAP, and CD68 in the CA1 region of the hippocampus (Zhu et al., 2018). 48 and 72 h of sleep deprivation produced a range of proinflammatory cytokines in the brain and blood of rats (Wadhwa et al., 2017). Sleep disruption-induced neuroinflammation may provide a convergent mechanism leading to opioid use (Cahill and Taylor, 2017).

The effects of sleep deprivation on drug use are mixed, and most evidence comes from experiments conducting acute but not chronic sleep deprivation. Six hours of sleep deprivation after conditioning and before testing did not affect morphine conditioned place preference (mCPP), but sleep deprivation after the test and before a priming session did disrupt morphine reward-related memory consolidation in CPP (Shi et al., 2011). 4 h of sleep deprivation during conditioning enhanced cocaine CPP at 3 and 8 mg/kg in mice, while 4 h sleep deprivation after conditioning enhanced CPP only at 8 mg/kg (Bjorness and Greene, 2020). Photoperiod shifting, which disrupts the normal sleep/wake cycle, blocked preference for food CPP in rats (McDonald et al., 2013). Acute sleep deprivation increased self-administration of sucrose in mice, dependent on basolateral amygdala to nucleus accumbens signaling (Wang et al., 2020). One month of constant light increased morphine preference in a two-bottle choice test compared to rats maintained on a standard light-dark schedule (Garmabi et al., 2016). Acute sleep deprivation increased the rate of lever pressing and hastened response for cocaine during progressive-ratio self-administration in low drug-taking rats (Puhl et al., 2009). 8 days of sleep restriction *via* the disk treadmill method did not affect low drug taking rats but high drug takers showed greater responding in fixed ratio and higher breakpoints in progressive ratio self-administration for cocaine (Puhl et al., 2013). Sleep restriction during the active/dark period, which condenses REM during the inactive/light phase decreased incubation of craving for cocaine, while increasing sleep fragmentation sped up the incubation of craving for cocaine (Chen et al., 2015). Overall, the effect of sleep deprivation on reward is mixed depending on the drug and on the type and length of sleep deprivation. More research in this area will help elucidate a better understanding of exactly how sleep deprivation contributes to drug reward.

Most individuals that have abused opioids report oral administration (McCabe et al., 2007; Katz et al., 2008). Therefore, we used a two-bottle choice method for administering morphine. Our method used a 0.2% saccharin solution in both the morphine and vehicle bottles, which generally produces a morphine preference in C57Bl6/J mice (Belknap et al., 1993), and avoids the confound of quinine influencing choice consumption more than morphine (Grim et al., 2018). We used an established model of CSS (Zhu et al., 2018) with non-invasive sleep (behavioral quiescence) recording and automated drinking sensors to assess the effect of prolonged sleep disturbance on both voluntary consumption and associative rewarding properties of morphine in male mice. This sleep deprivation did not affect corticosterone levels or affective behavior, but did increase Iba1 expression, in the hypothalamus. To confirm the impact of this sleep deprivation, we analyzed Iba1 expression, a marker of microglia, in the hypothalamus due to its role in mediating sleep/wake behavior (Saper et al., 2005), stress and endocrine response (Winsky-Sommerer, 2004), and morphine reward (Harris et al., 2007). We hypothesized that CSS would increase both voluntary consumption of and conditioned place preference for morphine. In contrast to our hypothesis, rested mice displayed morphine preference while CSS mice did not. There was no difference in morphine CPP between rested and CSS mice. These data represent, to the best of our knowledge, the first investigation of extended sleep deprivation as it relates to morphine consumption and reward in mice.

## Materials and Methods

### Mice

**FIGURE 1:**
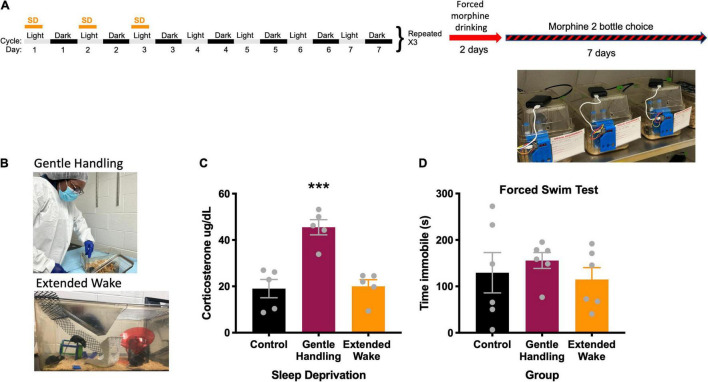
**(A)** Experimental design, **(B)** depiction of types of sleep deprivation, **(C)** radioimmunoassay of corticosterone comparing types of sleep deprivation, *** indicates *p* < 0.001. **(D)** Forced swim test comparing types of sleep deprivation.

Experiments were performed on male C57BL/6NTac mice and were 7 weeks old at the start of the experiment. Mice were housed individually for the sleep analysis and the following 2-bottle choice task (Figure 1A). A separate group of mice was housed in groups of 4 for the CPP experiment. A total of 20 animals (*n* = 10/group) were used for the 2-bottle choice experiments. Of the mice in the 2-bottle choice, 10 were housed in the sleep sensors, (*n* = 5 rested/5 CSS). In the CPP experiment, a separate cohort of 24 mice were used (*n* = 12 per group, rested vs. CSS), which all received morphine injections during CPP. Lights were maintained on a standard 12 h/12 h light cycle with lights on at 0700 h and off at 1900 h. CPP experiments occurred between 0900 and 1400 h. Animals were provided with *ad libitum* food and water access. All experiments were conducted in accordance with the NIH Guide for the Care and Use of Laboratory Animals and were approved by the University of Pennsylvania’s Institutional Animal Care and Use Committee (IACUC).

### Sleep Deprivation

We first evaluated two well-established methods of sleep deprivation, gentle-handling by direct touch and novel environment/extended wake (Figure 1B) in order to model CSS. To determine which sleep deprivation method would result in less stress, we measured serum corticosterone. In the gentle handling by direct touch method, mice were kept awake by an investigator constantly monitoring the state of the animal. When the animal appeared to be close to sleep, the investigator would open the cage and move the mouse around with a hand or paintbrush if necessary, which suppresses nearly all sleep (Franken et al., 1991), and increases corticosterone in an acute sleep deprivation (Kopp et al., 2006). Control mice in the gentle handling by direct touch experiment were in the same room but had no intervention from the experimenter. Extended wake was conducted by placing objects in a cage at different periods before the mouse falls asleep (Zhu et al., 2018). Objects included climbing wire, upside-down pencil holders, toys, and running wheels (Figure 1B). The extended wake paradigm was used for mice undergoing the 2-bottle choice and conditioned place preference studies. Here, mice were sleep deprived with the extended wake method for the first 8 h of the light cycle (0700–1500 h) on the first 3 days of 4 consecutive weeks. This method of long-term sleep deprivation *via* extended wake is referred to as CSS (See timeline in Figure 1A). Mice in the CSS group had standard bedding, water, and chow, with novel objects placed throughout the 8 h extended wake period. Rested control mice were placed in the same room for the first hour of the lights on period (0700–0800 h), when mice are typically awake, in order to expose them to the same room and stimuli without excessive sleep deprivation.

### Corticosterone

After a single acute sleep deprivation period of either gentle handling by direct touch or extended wake, trunk blood was collected for corticosterone assessment. Control mice were in the same room as the sleep deprivation mice but were left alone experimentally. Corticosterone was collected immediately at the end of the single acute sleep deprivation session at 1,500 h. All samples were collected from all groups at the same time on the same day. Corticosterone was not collected immediately after the 4 weeks of CSS because we did not want to interfere with subsequent behavioral experiments. Blood was collected in heparin coated tubes (Sarstedt Inc., prod. No. 16.443.100, Newton, NC) and was spun at 3000 RPM for 20 min. Corticosterone concentrations in plasma samples were measured using a commercial radioimmunoassay (MP Biomedicals, Solon OH; cat. no. 07120103). Standards and samples were analyzed in singlet by an experienced researcher, blind to treatment conditions, that produces low CVs with duplicates. Assay manufacturer guidelines were followed with the exception that volumes were halved for all reagents and samples; this method produces control sample values within the expected range and intra-assay coefficients of variation below 10%. Corticosterone concentrations are expressed as μg/dl.

### Forced Swim

To determine whether CSS resulted in depressive-like behaviors, we performed the forced swim test in rested and sleep deprived mice. The forced swim test was conducted on a separate group of mice that did not undergo CPP or 2-bottle choice. The FST was conducted after a single sleep deprivation session to determine the impact of sleep disruption on affective behavior that could confound interpretation of subsequent behavior experiments. Control mice were exposed to the same room as sleep deprived mice for the forced swim test but had no experimental manipulation. Plastic cylinders (23 cm × 14 cm diameter) were filled to 10 cm with 25°C water. Mice were placed in the cylinder and left for 6 min. Total immobility time for the entire 6 min period was scored by a trained observer blind to treatment conditions (Gundersen et al., 2013).

### Sleep Analysis

Sleep (behavioral quiescence) was monitored non-invasively using the COMPASS system (Continuous Open Mouse Phenotyping of Activity and Sleep Status) (Brown et al., 2017) in which homecages are fixed with infrared sensors on the center of the top of the cage (Figure 1A). Activity is generated continuously (100 ms increments 100 times for a 10s bin) and results in data binned every 10s with a score between 0 and 100 depicting quantification of movement in each of the 100 increments in the 10s bin. Continuous inactivity of more than 40 s in this system correlates with EEG measures of sleep at Pearson coefficients of 0.95 or greater (Brown et al., 2017). Periods of immobility (0 activity for more than 40 s) were converted to time sleeping in python and time sleeping was plotted and analyzed for the dark cycle and light cycle separately in GraphPad Prism.

### Two-Bottle Choice Test

Following the last day of the 4 weeks of CSS, mice underwent a forced administration of morphine in the drinking water (0.5 mg/mL morphine dissolved in 0.2% saccharin to neutralize the bitter taste of morphine) (Eacret et al., 2021). Pilot experiments showed that mice did not develop a preference to morphine without this 2-day morphine acclimation period (data not shown). After this 2-day forced exposure, mice were exposed to one bottle of 0.5 mg/ml morphine in 0.2% saccharin and one bottle of 0.2% saccharin alone. These liquids were in 15 mL conical tubes placed in a 3D printed lickometer with sensors under the sipper tubes to continuously monitor intake from each bottle (Godynyuk et al., 2019; Figure 1A). Mice were exposed to both morphine in saccharin and saccharin bottles for 7 days. Volume was measured manually every 24 h as well as continuously from the lickometer system and binned into average content preference every 12 h. Plots from the lickometer data were generated in SipperViz.py.^1^

### Conditioned Place Preference

Morphine conditioned place preference (mCPP), following 4 weeks of CSS was conducted on a separate cohort of mice in order to assess context-related associations of the rewarding properties of morphine (for review, see McKendrick and Graziane, 2020). Fifteen minute pre- and post-conditioning test sessions and eight daily 30-min conditioning sessions consisting of alternating side-pairings of morphine (10 mg/kg, s.c.) and saline (0.9% saline, s.c.) were used to determine conditioned place preference (Walters et al., 2005). Mice were acclimated to the mCPP behavioral room for 1 h on two separate days prior to the CPP pre-test following the end of the 4 weeks of sleep deprivation. During the pre-test session, mice were placed in a two-chambered place preference chamber (Med Associates, inc., St. Albans, VT) inside a sound-attenuated chamber (Med Associates, inc., St. Albans, VT) and allowed to explore both sides for 15 min (900 s). The amount of time spent on each side was recorded, and data were used to divide the animals into groups with similar biases on each side. Mice were conditioned daily for 8 days after the pre-test, with one side of the box exposed on odd pairing days and the other side exposed on even pairing days. Each group received morphine (10 mg/kg: morphine sulfate, NIDA Drug Supply, Research Triangle Park, NC) on one side and saline (0.9% sodium chloride) on the other. Drug-paired sides were randomized among groups. Locomotor activities for each conditioning session were measured using two consecutive beam breaks. One day after the last conditioning session, animals were allowed to explore freely between the two sides for 15 min (900 s), and time spent on each side was recorded. The preference score (time spent on the drug-paired side minus time in the saline-paired side on the postconditioning day minus the pre-conditioning day) was calculated for each mouse.

### Statistics

The statistical test was chosen based on the number and type of comparisons. For comparisons between 2 groups, *t*-tests were used, while a one-way ANOVA was used for groups of more than 2. For sleep studies, body weight, volume consumed, and locomotor activity over time, a two-way ANOVA with repeated measures by day was used. If a main or interaction effect occurred, Tukey’s *post-hoc* test was used for multiple comparisons. For continuous data, area under the curve with 95% confidence intervals (CI) were reported. *F*-tests were used to detect possible differences in variance between groups. The alpha value was set to 0.05 to denote statistical significance.

## Results

### Corticosterone, Sleep, and Body Weight Responses to Sleep Deprivation

We found that the extended wake method to prevent sleep was less stressful to mice compared to the gentle handling by direct touch method (Figure 1C). In the corticosterone assessment following both deprivation methods, there was a main effect of sleep deprivation [*F*(2, 12) = 19.67, *p* = 0.0002] (Figure 1C). Tukey’s *post-hoc* test revealed the gentle handling by direct touch sleep deprivation increased corticosterone compared to extended wake (*p* = 0.0005) and rested controls (*p* = 0.0004). There was no significant difference in immobility in the forced swim test following sleep deprivation [*F*(2, 15) = 0.4516, *p* = 0.6450] (Figure 1D).

**FIGURE 2:**
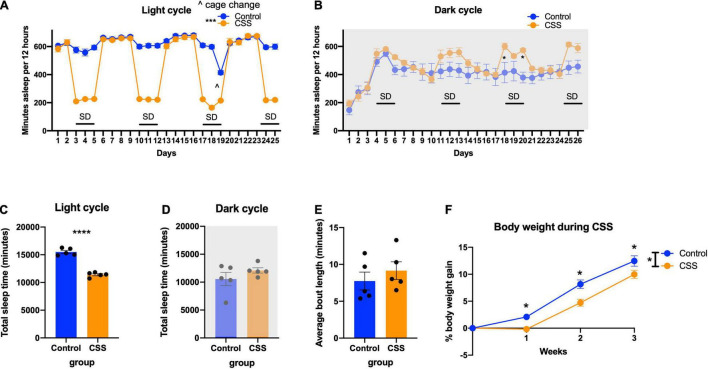
**(A)** Time sleeping per 12 h during the light (inactive) cycle each day during sleep deprivation. *** indicates *p* < 0.001. **(B)** Time sleeping per 12 h during the dark (active) cycle. * indicates *p* < 0.05 in Tukey’s *post-hoc* test. **(C)** Total time sleeping across the duration of the experiment in the light cycle. **** indicates *p* < 0.0001. **(D)** Total time sleeping across the duration of the experiment in the dark cycle. **(E)** Average bout length of sleep during the 4 weeks of sleep deprivation. **(F)** Body weight during the weeks of sleep deprivation. * indicates *p* < 0.05, ^∧^ indicates cage change.

During the light cycle, the sleep deprived mice got less sleep over the duration of the study [*F*(1, 8) = 145.7, *p* < 0.0001] (Figure 2A), and had significantly decreased total sleep (t = 12.07, df = 8, *p* < 0.0001) (Figure 2C). Tukey’s *post-hoc* test showed that there were no differences in sleep time during the light cycle on non-sleep deprivation days (*p* > 0.96). During the dark cycle, there was no main effect of sleep deprivation [*F*(1, 8) = 1.467, *p* = 0.2604), but there was a Day × CSS interaction [*F*(25, 200) = 5.696, *p* < 0.0001) (Figure 2B). Tukey’s *post-hoc* test revealed significant increases in recovery sleep on day 18 (*p* = 0.0260) and day 20 (*p* = 0.0165) (Figure 2B). There was no difference in total sleep time between rested mice and CSS mice in the dark cycle (t = 1.211, df = 8, *p* = 0.2604) (Figure 2D). There were no differences in the average bout length of sleep between rested and CSS mice throughout the study (t = 0.8246, df = 8, *p* = 0.4335) (Figure 2E).

CSS mice in the 2-bottle choice experiment gained less weight than rested control mice [*F*(1, 38) = 10.47, *p* = 0.0025] and showed a time x sleep interaction [*F*(3, 114) = 4.451, *p* = 0.0054] (Figure 2F). Tukey’s *post-hoc* test showed a significant difference between rested and CSS mice at weeks 1 (*p* = 0.0388), 2 (*p* = 0.0005), and 3 (*p* = 0.0191).

### Microglial Responses to Chronic Short Sleep

CSS mice showed increased Iba1 expression (positive cell counts per mm^2^) in the hypothalamus compared to rested control mice (*t* = 2.182, df = 16, *p* = 0.0443) (Supplementary Figures 1A–C).

### 2-Bottle Choice

**FIGURE 3:**
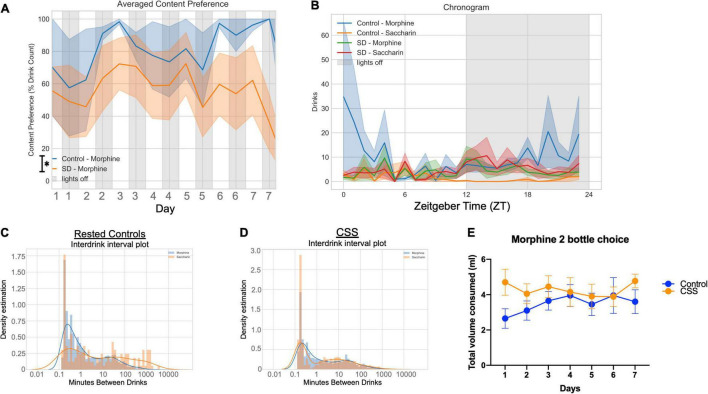
**(A)** Average content preference over 7 days between rested and chronic short sleep (CSS) groups, plotted in 12-h bins. **(B)** Averaged chronogram showing drinks per hour of circadian time for all 7 days. **(C)** Interdrink interval between morphine and saccharin for the rested control group. **(D)** Interdrink interval between morphine and saccharin for the chronic short sleep group. **(E)** Total volume consumed, measured manually, of both morphine and saccharin combined for both the rested and sleep deprived groups.

Rested control mice during the 7 days of 2-bottle choice had increased area under the curve (total area 15,203, 95%CI 13,013–17,394) for preference percentage compared to CSS mice (total area 9,554, 95%CI 6,864–12,244) (Figure 3A) indicating a greater preference for morphine in non-sleep deprived mice. Of interest, rested and CSS mice drank similar total volumes [*F*(1, 19, = 1.907), *p* = 0.1834] (Figure 3E). The preference pattern and volume measurements were manually verified every 24 h. The average grouped chronogram shows that over the 7-day experiment, the rested mice drank more morphine during the late part of the dark/active period into the early part of the light/inactive period (Figure 3B). Graphs of drink counts over 7 days further display circadian information and show less light cycle morphine drinking in control compared to CSS (Supplementary Figures 2A,B). Rested and CSS mice have a different pattern of inter-drink intervals with respect to morphine (Figures 3C,D), interpreted as rested controls drink more actively from the morphine bottle, thereby showing a higher frequency of drinks with lower time between drinks.

**FIGURE 4:**
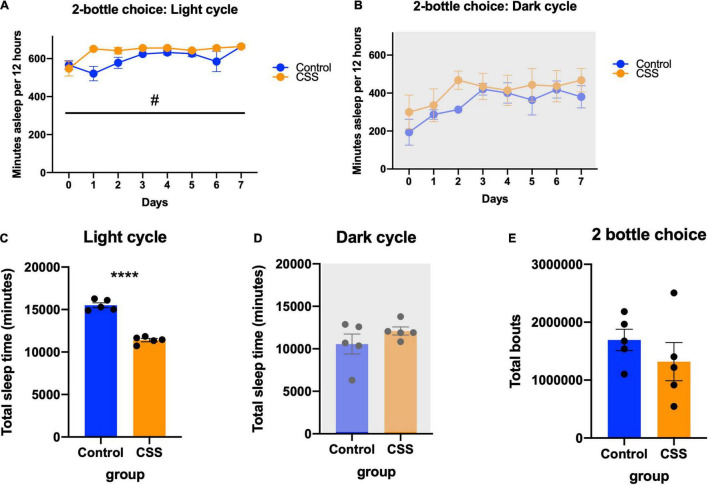
**(A)** Minutes asleep per 12 h during the light (inactive) cycle during the 2-bottle choice experiment. ^#^Denotes significant main Day × CSS interaction effect **(B)** minutes asleep per 12 h during the dark (active) cycle during the 2-bottle choice experiment. **(C)** Average bout length compared between rested and sleep deprived mice during the 2-bottle choice experiment. **(D)** Maximum bout length compared between control and CSS during the 2-bottle choice experiment. **(E)** Total bouts of sleep during the 2-bottle choice experiment. **** indicates *p* < 0.0001.

Sleep during the 2-bottle choice test was analyzed, and during the light cycle there was a trend toward increased sleep during the light cycle in CSS mice [*F*(1, 8) = 5.180, *p* = 0.0524], and a significant day x sleep interaction [*F*(7, 56) = 2.544, *p* = 0.0240] (Figure 4A). In the dark cycle during the 2-bottle choice there was no difference between rested and CSS mice [*F*(1, 8) = 1.402, *p* = 0.2704] and no day x sleep interaction [*F*(8, 64) = 0.6251, *p* = 0.7537] (Figure 4B). There was no significant difference in average sleep bout length between rested and CSS mice during the 2-bottle choice period (*t* = 1.690, df = 8, *p* = 0.1294) (Figure 4C). There was no significant difference in maximum bout length (*t* = 1.587, df = 8, *p* = 0.1511) (Figure 4D) or total bouts (*t* = 0.9921, df = 8, *p* = 0.3502) (Figure 4E).

### Conditioned Place Preference

**FIGURE 5:**
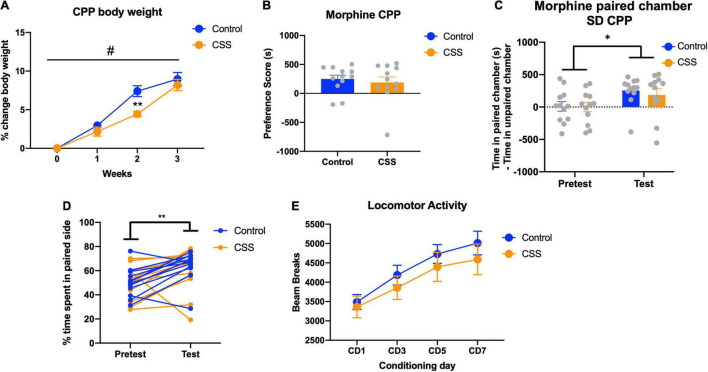
**(A)** Body weight for the animals in the CPP cohort during the 4 weeks of sleep deprivation. ** indicates *p* < 0.01. **(B)** Preference score for CPP, defined by time in the morphine-paired chamber on the post-conditioning test day of CPP minus time in the morphine-paired chamber on the pretest. **(C)** Time in the morphine paired chamber minus time in the saline paired chamber, for both pretest and test. **(D)**% of time spent on the morphine-paired side of the CPP chamber on both the pretest and posttest, repeated measures by animal. **(E)** Locomotor activity from the 4 days of conditioning with morphine. * indicates *p* < 0.05, # indicates significant main week × CSS interaction effect.

Mice in the sleep deprivation-CPP study, which was conducted after 4 weeks of CSS, had a non-significant trend toward decreased body weight [*F*(1, 22) = 3.743, *p* = 0.0660] (Figure 5A). In this experiment there was a week x CSS interaction effect [*F*(3, 66) = 4.637, *p* = 0.0053], and Tukey’s *post-hoc* test revealed a significant difference in body weight between rested and CSS at week 2 (*p* = 0.0065). Both groups displayed a preference for the morphine paired side of the chamber [*F*(1, 22) = 6.452, *p* = 0.0187] (Figure 5C), with no significant differences in preference score (*t* = 0.5258, df = 22, *p* = 0.6043) (Figure 5B). A 2-way repeated measures ANOVA revealed a morphine preference between pretest and test [*F*(1, 22) = 13.80, *p* = 0.0012] and an effect of subject [*F*(22, 22) = 2.247, *p* = 0.0320] (Figure 5D). Both groups showed a locomotor sensitization to morphine, with ambulation increasing over the 4 conditioning days [*F*(2.421, 50.83) = 29.43, *p* < 0.0001] (Figure 5E). There was no effect of CSS on locomotion during the conditioning days [*F*(1, 21) = 0.6605, *p* = 0.4255] (Figure 5E).

## Discussion

Here, we provide the first study of long-term sleep disruption on morphine intake and reward in mice. Our long-term CSS paradigm does not increase corticosterone levels and does not change immobility in the forced swim test. There does appear to be somewhat of a bimodal distribution in the FST, highlighting individual differences in response to sleep deprivation, however the group size is not sufficient to reliably generate two distinct subpopulations. Individual differences with respect to sleep deprivation could affect recovery sleep as well as preference in the 2-bottle choice and CPP experiments. Our sleep deprivation period restricts sleep by 25% during the light cycle over the total time course of the experiment, and as NREM and REM total sleep time are associated with corticosterone regulation more strongly in the light cycle than the dark cycle (Nollet et al., 2019), sleep disruption during the normal inactive period of the animal would be more robust than sleep disruption during the active period. The lack of stress response and no changes in the FST allows us to isolate the effects of lack of sleep vs. confounds such as increased corticosterone and altered affective behaviors, which could themselves influence morphine consumption and/or conditioned reward, as stress changes the rewarding properties of drugs of abuse and causes changes in reinstatement (Will et al., 1998; Mantsch et al., 2016). That CSS mice had increased recovery sleep in the subsequent dark period after sleep loss supports effective sleep deprivation in this study, as does increased Iba1 expression in the hypothalamus.

Following exposure to CSS, we assayed two different methods of morphine reward, voluntary intake and paired association. Based on the clinical data, we hypothesized that CSS would increase vulnerability to opioid use- in our model reflected by increased morphine preference in the 2-bottle choice and increased time in the morphine-paired chamber in the CPP test. However, we found that chronic sleep disruption prevents morphine preference and does not affect conditioned reward. One interpretation of lower morphine preference in the sleep deprivation group is that less morphine is required for reward. Another is that morphine itself is less rewarding, or even becomes aversive. The CPP test was conducted to determine whether the latter interpretation is valid, yet we found that the rewarding associative properties of morphine are the same between rested and CSS mice. Of note, the dose of morphine tested in the CPP paradigm (10 mg/kg) is comparable to the dose of morphine ultimately consumed in the 2-bottle choice test; mice drank 4 mL total per day, 60% of which is morphine, which calculates to approximately 15 mg/kg/day based on a 25 g mouse. While this study examined the impact of extended sleep disruption on morphine reward, future studies should examine the impact of opioid use on sleep quality.

Advantages of the oral self-administration paradigm are that it allows us to examine how morphine exposure affects sleep without any injections or surgery, typically required in intravenous-self administration models. This is particularly important as acute opioids administered *via* injection can disrupt sleep but long term studies of the effects of opioids on sleep are rare (Coffey et al., 2016; Eacret et al., 2020). Further, the lickometer system used in combination with the COMPASS system allowed us to observe morphine intake throughout the circadian time course. These automated, continuous data-generating apparatuses provide for a non-biased, non-invasive, and more ethologically relevant analysis than traditional methods, which is actively promoted in the field of sleep and addiction research (Depoy et al., 2017).

Limitations of this study are that memory was not assayed in response to sleep deprivation. The CSS paradigm has been shown to decrease spatial object preference in B6 mice (Owen et al., 2021), thus, from this assay alone we cannot exclude that the animals’ ability to discern left from right and therefore morphine from saccharin, could have been influenced by sleep loss. The morphine and saccharin bottles remained on the same side during the 2-bottle choice experiment to decrease the requirement of memory in the task. Morphine has a ∼3 h half-life, so had we switched the sides of morphine and saccharin bottles it could have disrupted the animal’s true preference. In pilot experiments we found that rested mice do not develop a preference without the initial 2 days of forced morphine exposure and switching sides of the morphine or saccharin bottles disrupts preference for 1–2 days. The CSS mice did, however, show place preference for morphine and thus do demonstrate intact spatial memory at least for this assay. Another limitation is that these studies were conducted in male mice. Future studies should determine whether disrupted sleep affects vulnerability to opioid use differentially according to sex. Further, enriched environment and exercise have been shown to be neurogenic and antidepressant (Hattori et al., 2007; Kobilo et al., 2011; Nicolas et al., 2015), and our extended wake paradigm does provide an enriched environment of sorts. There was no difference in the forced swim test after extended wake, a behavioral test previously used as a screen for antidepressant efficacy (Porsolt et al., 1978; Abelaira et al., 2013), however, as with corticosterone measurements, this was conducted after a single sleep deprivation session, not following the full 4 weeks of CSS. This provided evidence that the method of sleep deprivation was not acutely stressful. Given that we do not have FST or corticosterone data after CSS, we cannot rule out that the continued and extended sleep disruption is stressful in these particular mice. However, the same paradigm of 4 weeks of CSS does not produce changes in corticosterone (Zhang et al., 2014). Sleep in this study was defined by behavioral quiescence from the non-invasive COMPASS system. While this system can interpret sleep with an accurate correlation to EEG measures of sleep (Brown et al., 2017), given that behavioral quiescence is defined as 40s + of immobility and we show ∼600 m sleep in the light cycle and ∼400 m sleep in the dark cycle, it is possible that behavioral quiescence overestimates sleep as the animal could be awake but immobile. This system was sensitive enough to show increases in recovery sleep in the dark cycle following the daytime sleep deprivation, though it lacks the ability to capture spectral power to interpret sleep intensity. Sleep deprivation can increase sleep intensity and EEG methods would be necessary to interpret sleep differences in this way (Borbély et al., 1984).

Overall, we show that recovery sleep after CSS blocks morphine preference in a voluntary intake context but not in a paired association context. This was a surprising result to us, as in humans it appears that short sleep promotes vulnerability for drug use. For that reason, we conducted an additional experiment (CPP) to either verify or inform this phenotype. We found that while CSS blocks morphine preference in a voluntary intake model, there was no difference in mCPP after CSS. It is possible that our preclinical model does not accurately reflect the human condition, in which case it is still important to know how CSS affects opioid intake in mice. It is also possible that sleep disruption is dose-dependent, and different durations of sleep disruption than that used in this experiment could provide different results. While these results are preliminary and more work needs to be done in order to elucidate the neurobiological mechanisms underlying sleep disruption-induced changes in opioid intake, this study marks a step toward creating a foundation for this realm of research. Despite correlational epidemiological findings which have been used to link sleep dysfunction and opioid use, our study found no detrimental impact of CSS on opioid reward. Future studies should focus on elucidating how to use sleep as a symptom or phenotypic marker of opioid use disorder or withdrawal. There could exist the possibility to target sleep disruption in order to ameliorate opioid use and symptoms.

## Data Availability Statement

The raw data supporting the conclusions of this article will be made available by the authors, without undue reservation.

## Ethics Statement

The animal study was reviewed and approved by the University of Pennsylvania Institutional Animal Care and Use Committee.

## Author Contributions

DE and JB conceptualized the experimental design, with input on sleep deprivation from SV. DE and CL performed the behavioral methodology, investigated, validated the data, and carried out the analysis. JC and SC performed the radioimmunoassay. JB provided the funding acquisition and resources. DE wrote the initial draft of the manuscript. SV and JB reviewed and edited the manuscript. All authors contributed to the article and approved the submitted version.

## Conflict of Interest

The authors declare that the research was conducted in the absence of any commercial or financial relationships that could be construed as a potential conflict of interest.

## Publisher’s Note

All claims expressed in this article are solely those of the authors and do not necessarily represent those of their affiliated organizations, or those of the publisher, the editors and the reviewers. Any product that may be evaluated in this article, or claim that may be made by its manufacturer, is not guaranteed or endorsed by the publisher.

## Footnotes

1
https://github.com/earnestt1234/SipperViz
