# Supplementary material for: Chronic Sleep Deprivation Blocks Voluntary Morphine Consumption but Not Conditioned Place Preference in Mice
Source: Front Neurosci. 2022 Feb 17;16:836693. doi: 10.3389/fnins.2022.836693 (PMC8892254; doi:10.3389/fnins.2022.836693)
Supplement: Supplementary file 1 [file Data_Sheet_1.pdf]

Immunohistochemistry

Mice were sleep deprived parallel with mice used in the 2-bottle choice experiment (9 days after the last sleep deprivation session) but not exposed to morphine. Mice were deeply anesthetized with sodium pentobarbital (50mg/kg i.p.) and perfused with cold 0.01M phosphate-buffered saline (PBS) and 4% paraformaldehyde (PFA). Brains were removed and placed in a 15mL conical tube with 4% PFA overnight for 24h then switched to 30% sucrose and stored at 4°C. Brains were sliced at 14 microns on a cryostat (Cryostar NX-50, Thermo Scientific, Waltham, MA). Sections were mounted onto microscope slides (Superfrost Plus, ThermoFisher, cat. no. 22-037-246, Waltham, MA) and washed 3 x 10m in PBS. Slides were blocked for 1h in 10% normal donkey serum (NDS) in PBS with 0.1% Triton-X then incubated overnight at 1:500 with rabbit anti-Iba1 (Wako, cat. no. 019-19741). The next day, slides were washed 3 x 5 minutes and incubated with AlexaFluor 488 goat anti-rabbit at 1:1000, protected from light (Life Technologies, cat. no. A-11008). Slides were washed 3 x 5 minutes with PBS and were coverslipped with DAPI Fluoromount (SouthernBiotech, cat. no. 0100-20, Birmingham, AL). Three separate images across the anterior to posterior axis of the hypothalamus were acquired per animal and overlayed on the hypothalamus to generate a region of interest based on the Allen Brain Atlas. Iba1 positive cells were counted and quantified with Fiji (ImageJ) cell counter tool, and the 3 sections were averaged to get one count of Iba1 positive cells per mm^2 per animal, by an experimenter blind to treatment conditions until the end of quantification.

Figure 1

a) Representative image of Iba1 staining in the hypothalamus of the rested and the b) chronic short sleep (CSS) group, and c) quantification of Iba1+ cells per mm^2. * indicates $p < 0.05$.

Figure 2

a) 7-day graph of drink counts (represented on the y-axis from information from photosensor breaks below the respective sipper tubes) in the 2-bottle choice experiment from the rested control group and b) the chronic short sleep (CSS) group.
