# Supplementary figures and images for: Chronic Sleep Deprivation Blocks Voluntary Morphine Consumption but Not Conditioned Place Preference in Mice
Source: Front Neurosci. 2022 Feb 17;16:836693. doi: 10.3389/fnins.2022.836693 (PMC8892254; doi:10.3389/fnins.2022.836693)

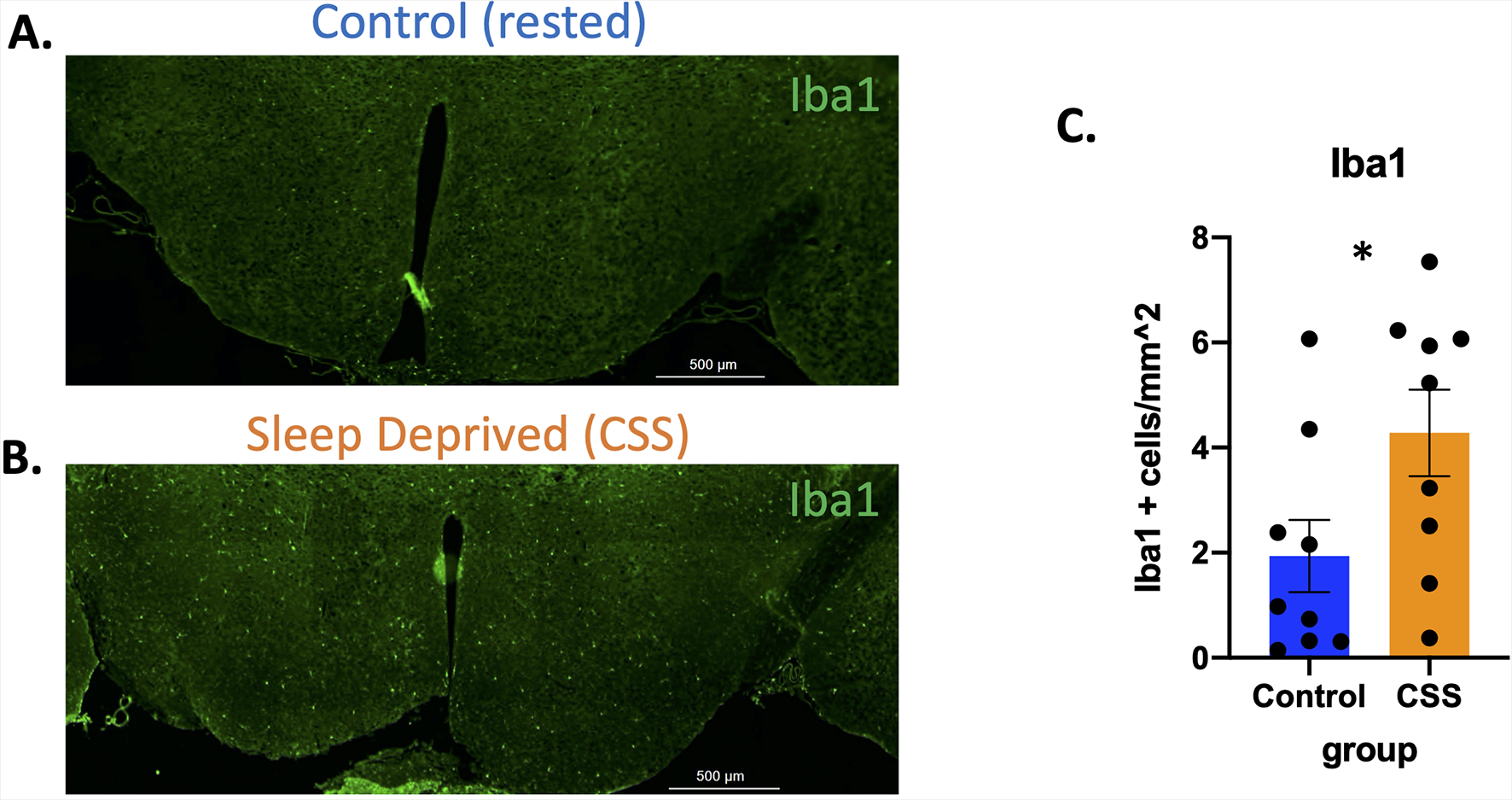

Supplement: Supplementary file 2 [file Image_1.tiff]

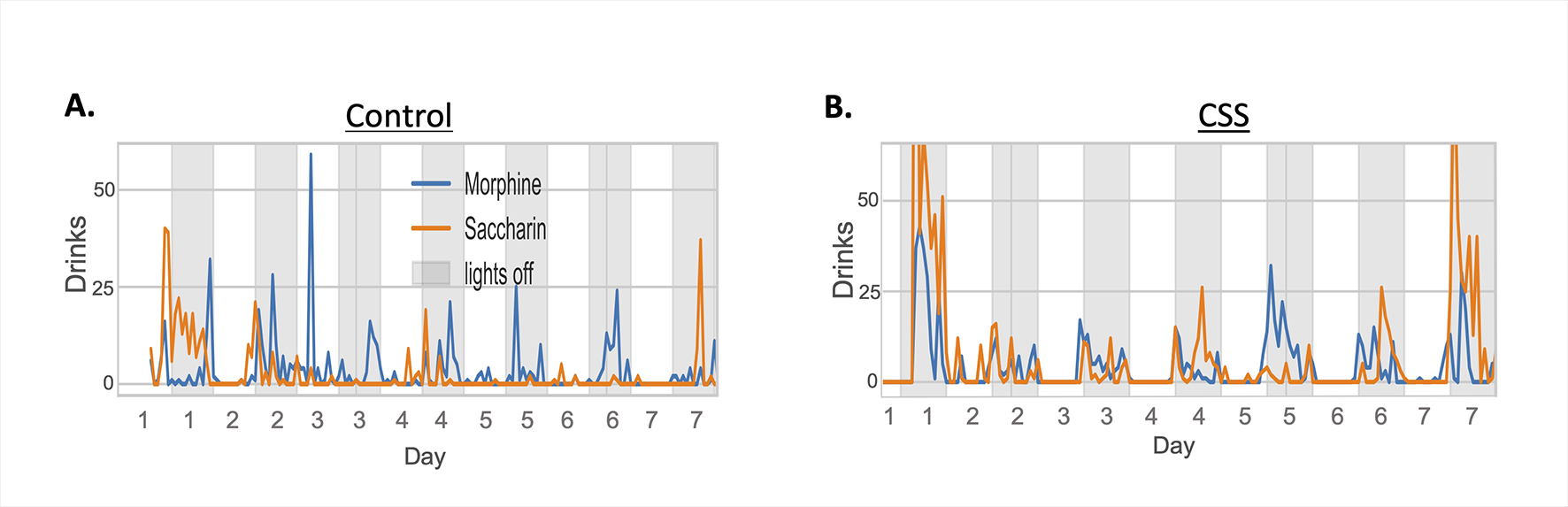

Supplement: Supplementary file 3 [file Image_2.TIFF]
